# Increased frequency of circulating Tc22/Th22 cells and polyfunctional CD38^−^ T cells in HIV-exposed uninfected subjects

**DOI:** 10.1038/srep13883

**Published:** 2015-09-08

**Authors:** Luanda M. S. Oliveira, Josenilson F. Lima, Cesar A. C. Cervantes, Jorge S. Casseb, Marcelo Mendonça, Alberto J. S. Duarte, Maria N. Sato

**Affiliations:** 1 Laboratory of Dermatology and Immunodeficiencies, LIM-56, Department of Dermatology, Tropical Medicine Institute of São Paulo, University of São Paulo, São Paulo, Brazil; 2 Ambulatory Service of the Secondary Immunodeficiency Clinic of the Clinical Hospital, University of São Paulo Medical School, São Paulo, Brazil; 3 Emílio Ribas Infectious Diseases Institute in São Paulo, Brazil

## Abstract

Some individuals are resistant to HIV-1 infection despite repeated exposure to the virus, suggesting the presence of a complex antiviral response. Innate factors like IL-22 exert gut mucosal protection and polyfunctional T cells have been associated with low progression in HIV infection; therefore, we evaluated the frequencies of CD4+ and CD8+ T cell-secreting cytokines, including Tc22/Th22 cells and polyfunctional T cells in HIV-1-exposed uninfected individuals (EUs), their HIV-1-infected partners and healthy controls. EUs exhibited an increased frequency of p15 Gag CD4+ IL-22+ secreting T cells, whereas HIV-infected partners demonstrated a high frequency of CD4+ IL-17+ T cells in response to p24. Similar responses of Th22 and Tc22 cells to Gag peptides and Staphylococcal enterotoxin B (SEB) stimulation were detected in the serodiscordant couples. However, polyfunctionality in HIV subjects was associated with an HIV Gag response of CD38+ T cells, whereas polyfunctionality for EUs was induced upon SEB stimulation by CD38- T cells. EUs demonstrated the presence of Tc22/Th22 cells and polyfunctional CD38- T cells with a low activation profile. These data suggest that SEB-induced polyfunctional CD4+ and CD8+ T cells together with Tc22/Th22 cells in EU individuals can provide an immunological advantage in the response to pathogens such as HIV-1.

Currently, approximately 35 million people are infected with human immunodeficiency virus type 1 (HIV-1) worldwide^1^. Susceptibility to HIV-1 infection differs among individuals, and a small number of subjects that are exposed to HIV-1 do not become infected; these individuals are called HIV-exposed uninfected individuals (EUs)^2^. These EUs belong to various risk groups, such as sex workers, health professionals, hemophiliacs who receive HIV-1-infected blood, drug users, children of HIV-1-infected mothers and individuals in stable relationships with partners that are HIV-1-infected^2^,^3^,^4^.

Some factors contribute to the decreased susceptibility to HIV-1 infection observed in EUs, including genetic homozygosity for the deletion of 32 base pairs of the CCR5 chemokine receptor^5^, antiviral innate immunity factors^6^, secretion of chemokines and cytokines, the activity of natural killer cells^4^, IgA or IgG^3^ antibody production against envelope proteins^7^,^8^, responses of T cells that are specific for HIV-1^9^, cytotoxic responses of polyfunctional CD8 T cells^10^ and low-level activation of CD4 T cells^11^.

In addition to innate anti-HIV-1 factors, IL-22 has been described as inducing the production of acute phase proteins that are able to inhibit HIV infection *in vitro*^12^. The functionality of IL-22 is associated with gut mucosal protection during HIV infection^13^,^14^. However, decreased IL-22 secretion in the genital mucosae and in the blood compartment induced by staphylococcal enterotoxin stimulation has been described in female EU sex workers^15^. In addition, EU sex workers in Kenya have demonstrated decreased IL-17 production in both blood and genital mucosal cells, indicating that resistance to infection could be related to a decreased proinflammatory response^15^. The cytokines IL-17 and IL-22 are secreted by several cells, including Th17 and Th22 cells, and by a subset of CD8+ T cells, termed Tc22 cells, that have not yet been investigated in EU individuals.

EU subjects are characterized by reduced immune activation with decreased CD4+ T cell frequency and the expression of activation markers such as HLA-DR, CD38 and CD70^11^. CD4+ T cells in EU sex workers demonstrate apparent reduced activation that may contribute to host resistance to HIV-1^16^.

The role of CD8+ T cells in the co-production of cytokines and cytotoxic molecules is important for viral control in HIV-1 infection^17^. A high frequency of HIV-specific polyfunctional CD8+ T lymphocytes in the blood of patients who control the virus, such as long-term non-progressors or elite controllers, is associated with spontaneous control of viral replication^18^,^19^.

Furthermore, the capacity of a specific subset of CD4+ T cells to secrete several cytokines to control viral replication and clearance, designated polyfunctional cells, represents another resistance mechanism for HIV-1 infection^20^. Additionally, CD8+ T cells in EU individuals produce a cytotoxic and polyfunctional response specific to HIV-1 that might confer protection against HIV-1 infection^10^.

In this study, we investigated IL-22- and IL-17-secreting T cell profiles in serodiscordant couples for HIV-1 in response to the p17, p24 and p15 regions of HIV-1 Gag protein or Staphylococcal enterotoxin B (SEB). We observed increased frequencies of Th22 and Tc22 cells in both HIV and EU individuals compared to the healthy control (HC) group. Interestingly, although we identified some immunological similarities between EU and HIV-infected partners, a distinct polyfunctional T cell profile was evident in EU individuals.

## Results

### Frequency of circulating CD4+ T cells secreting IL-17a or IL-22 in response to Gag in serodiscordant couples

IL-17 and IL-22 protein levels were significantly reduced in EU female sex workers in Kenya compared to controls^15^. However, the frequency of Gag response by CD4+ or CD8+ T cells secreting IL-17 and IL-22 in EU is unknown. We enrolled serodiscordant couples (EU and HIV-infected) and uninfected individuals to assess the presence of CD4+ and CD8+ T cells secreting IL-17a and IL-22 among PBMCs upon stimulation with HIV Gag peptides and SEB using flow cytometry. Because the presence of p24 Gag-induced CD4+ T cells is associated with nonprogression in HIV-1 infection^21^ we decide to evaluate the 3 pools of HIV-Gag peptides comprising p17, p24 and p15, respectively. The gating strategy is shown in Supplementary Figure 1.

**Figure 1:**
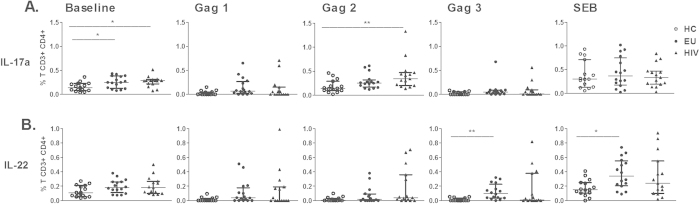
Circulating Gag*-s*pecific and SEB-induced CD4+ T cells secreting IL-22 in EU subjects. PBMCs from healthy controls (HC, n = 15), EUs (n = 16) and HIV-infected individuals (n = 15) were cultivated with medium (baseline), HIV Gag peptide pools [Gag1 (p17), Gag2 (p24), and Gag3 (p15)], or SEB for 6 h and Brefeldin A for 4 h. CD4+ T cells secreting IL-17a (A) and IL-22 (B) were assessed using flow cytometry. Frequency of CD4+ T cells was subtracted from baseline. The results are expressed as medians and IQRs. *p ≤ 0.05 and **p ≤ 0.01, adjusted for multiple comparisons.

An increased frequency of CD4+ IL-17+ T cells at baseline level was detected in both EU and HIV-infected subjects compared to the HC group (Fig. 1A). Only HIV-1-infected individuals demonstrated an expansion of CD4+ IL-17+ T cells induced by Gag2 (p24) stimulation. The frequency of CD4+ T cells secreting IL-22 in response to Gag3 (p15) peptide stimulation and for SEB was markedly increased in EUs compared to HC individuals (Fig. 1B).

**Figure 2:**
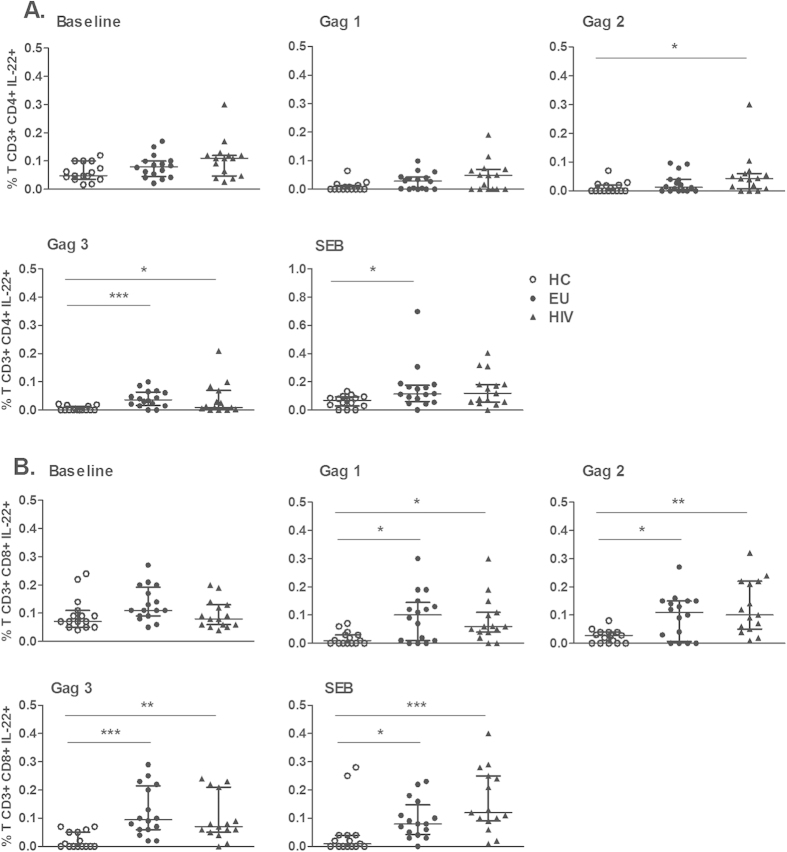
Presence of circulating Th22 and Tc22 cells in EUs and HIV-1-infected subjects. PBMCs of HCs (n = 15), EUs (n = 16) and HIV-infected individuals (n = 15) were cultivated with medium (baseline), HIV Gag peptide pools [Gag1 (p17), Gag2 (p24), and Gag3 (p15)], or SEB for 6 h and Brefeldin A for 4 h. Th22 and Tc22 cell frequencies were obtained by excluding IFN-γ and IL-17a. (**A**) Th22 and (**B**) Tc22 cell frequencies were assessed using flow cytometry. Frequencies of Th22 or Tc22 cells were subtracted from baseline levels. The results are shown as medians and IQRs. *p ≤ 0.05, **p ≤ 0.01 and ***p ≤ 0.001, adjusted for multiple comparisons.

Next, to verify whether the IL-22 response of CD4+ T cells was related to Th22 cells, we evaluated IL-22 expression, excluding IFN-γ- and IL-17 secreting cells in CD4+ T cells as previously described, to identify Th22 cells. The gating strategy is shown in Supplementary Figure 2. Curiously, p15-specific stimulation induced increased frequencies of Th22 cells in both HIV-1 and EU subjects compared to the HC group (Fig. 2A), and p24 induced Th22 expansion in HIV-1 individuals (Fig. 2A). Again, an increased frequency of Th22 cells was induced only in the EU group with SEB stimulation.

The frequencies of CD8+ T cells secreting IL-22 (Tc22) were increased in the EU and HIV-1 groups for stimulation with the Gag3 peptide pools and for SEB compared to the HC group (Fig. 2B). This finding indicates that in the EU group, CD8+ IL-22+ IFN-γ- IL-17- T cells have a broad Gag-induced response compared to CD4+ T cells.

The cytotoxic profiles of CD4+ and CD8+ T cells were evaluated based on the expression of two degranulating markers, CD107a and granzyme B. CD4+ CD107a+ T cells and CD8+ granzyme B+ T cells were detected at increased baseline levels or upon SEB stimulation in HIV-infected individuals compared to the HC group (Supplementary Figure 3). Moreover, no differences in the frequencies of CD4+ T cells secreting IL-17 or IL-22 were detected when HIV-1-infected individuals were analyzed based on their CD4 T cell counts or on detectable VLs; the same findings were observed in the respective EU partners.

These data demonstrate that EU individuals expressed higher frequencies of Th22 and Tc22 cells in response to Gag and SEB stimulation.

### Polyfunctional CD4+ and CD8+ T cells induced by Gag and SEB in EU subjects

Next, we verified the polyfunctional profile of CD4+ T cells that secrete IFN-γ, IL-2, IL-17a, IL-22 and MIP-1β induced by Gag and SEB stimulation in EUs and HIV-infected individuals. The gating strategy to detect simultaneous cytokine production in CD4+ T cells is shown in Supplementary Figure 4. Because the Boolean strategy generates several combinations, we show here only those data that yielded significant differences.

**Figure 3:**
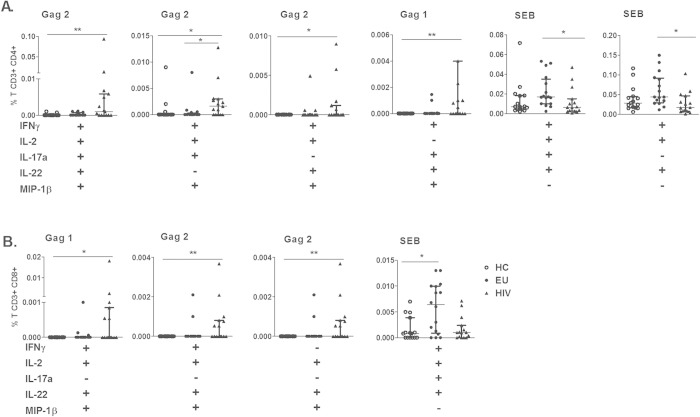
SEB-induced polyfunctional CD4+ and CD8+ T cells as features of EUs. The simultaneous secretion of IFN-γ, IL-2, IL-17a, IL-22 and MIP-1β by CD4+ (**A**) and CD8+ (**B**) T cells induced by Gag1 (p17), Gag2 (p24), or SEB from HC (n = 15), EU (n = 16) and HIV (n = 15) individuals was assessed using flow cytometry. Frequencies of CD4+ and CD8+ T cells were subtracted from baseline values. Bars represent the medians and IQRs. *p ≤ 0.05 and **p ≤ 0.01, adjusted for multiple comparisons.

A polyfunctional CD4+ T cell response to Gag stimulation was detected only in the HIV-infected group (Fig. 3). Increased frequency of CD4+ T cells secreting 5 cytokines were detected in response to Gag2 stimulation, or 4 cytokines in combination induced by Gag2 (excluding IL-22 or IL-17) and Gag1 (excluding IL-2) stimulation (Fig. 3A). In contrast, EU subjects demonstrated a polyfunctional response compared to the HIV-infected group only in response to SEB stimulation, for example, the secretion of 4 cytokines (IFN-γ, IL-2, IL-17a, and IL-22) or a combination of 3 cytokines (including IFN-γ, IL-2 and IL-22).

In the HIV-1 group, a polyfunctional CD8+ T cell response consisting of a combination of 4 cytokines, IFN-γ, IL-2, IL-22, and MIP-1β, was detected in response to Gag1 and Gag2 stimulation as well as a response consisting of IL-2, IL-22, and MIP-1β (Fig. 3B). Similarly CD4+ T cells, a polyfunctional CD8+ T cell response was induced by SEB stimulation in the EU group compared to the HC group.

To verify whether the CD4+ T cell response to HIV Gag induction could be related to the expression of chronic activation markers, we analyzed IFN-γ, IL-2, IL-17a, IL-22 and MIP-1β secretion in the presence of CD38 expression in CD4+ T cells. The gating strategy is shown in Supplementary Figure 4.

**Figure 4:**
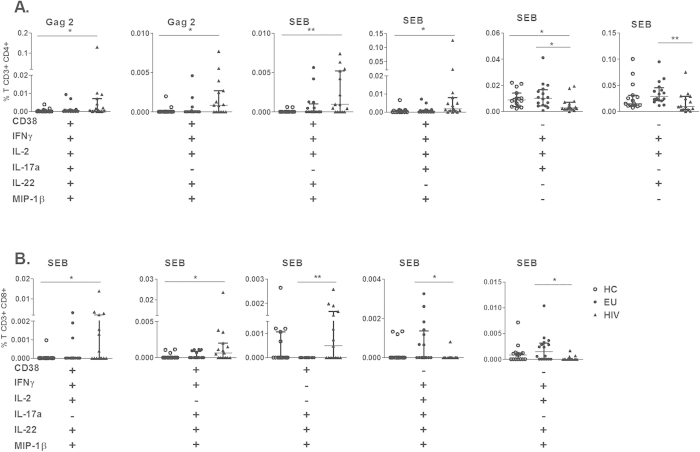
Distinct polyfunctionality profiles of CD4+ and CD8+ T cells based on CD38 expression in EUs and HIV-infected subjects. The simultaneous secretion of IFN-γ, IL-2, IL-17a, IL-22, and MIP-1β by CD4+ (**A**) and CD8+ T (**B**) cells expressing CD38 or not expressing CD38, induced by Gag1 (p17), Gag2 (p24), Gag3 (p15) or SEB, from HCs (n = 15), EUs (n = 16) and HIV-infected individuals (n = 15) was assessed using flow cytometry. (**C**) Pie chart representations of the capacity of CD4+CD38+ and CD4+CD38- T cells to secrete 5, 4 or 3 cytokines (IL-17a, IL-22, IFN-γ, MIP-1β and IL-2) induced by SEB. Frequencies of CD4+ and CD8+ T cells were subtracted from baseline values. Bars represent the medians and IQRs. *p ≤ 0.05 and **p ≤ 0.01, adjusted for multiple comparisons.

We observed that polyfunctional CD4+ CD38+ T cell Gag-response was only generated in the HIV group (Fig. 4A). Increased frequency of CD4+CD38+ cells secreting a combination of 5 cytokines in response to Gag2 was verified, and a combination of 3 cytokines, specifically IL-2, IL-22 and MIP-1β, in response to Gag2 stimulation was observed compared to HC subjects. Interestingly, a SEB-induced polyfunctional CD4+ CD38+ T cell response consisting of a combination of 4 (IFN-γ, IL-2, IL-22, and MIP-1β) or 3 (IFN-γ, IL-17a, and MIP-1β) cytokines was observed in the HIV group.

However, polyfunctional CD4+ CD38- T cell response was detected only in the EU group in response to SEB, consisting of 2 combinations of 3 cytokines (IFN-γ, IL-2, and IL-17a or IFN-γ, IL-2, and IL-22), compared to the HIV group (Fig. 4A).

Figure 4B shows that HIV-infected subjects demonstrated polyfunctional CD8+ CD38+ T cells in response to SEB stimulation, as evidenced by the secretion of 2 combinations of 4 cytokines (IFN-γ, IL-2, IL-22, and MIP-1β or IFN-γ, IL-17, IL-22, and MIP-1β) or a combination of 3 cytokines (IL-17, IL-22, and MIP-1β). However, in the absence of CD38, only the EU group developed polyfunctional CD8+ T cells inducible by SEB, secreting a combination of 4 or 5 (IFN-γ, IL-2, IL-17, IL-22 and MIP-1β) cytokines (Fig. 4B).

Our findings indicated a differential polyfunctional CD4+ T cell response and a CD8+ T cell response independent of stimulation. Polyfunctionality of CD4+ and CD8+ T cells induced by Gag was observed in CD38+ T cells in HIV-infected individuals, whereas in EU partners, the polyfunctionality was in CD38- T cells induced by nonspecific stimulation.

## Discussion

Here, we evaluated IL-22-secreting cells and HIV-specific or SEB-induced polyfunctional CD4+ and CD8+ T cells in EU subjects. Our results showed an expansion of circulating Gag response of Th22 and Tc22 in EU subjects. Importantly, we found that polyfunctional CD4+ and CD8+ T cells in EU subjects were evident only upon SEB stimulation, whereas the polyfunctional T cell responses in HIV-infected partners were induced by Gag. Moreover, in EU subjects, CD4+ T cell polyfunctionality occurred in the CD38- cells, in contrast to in the HIV partners. The findings revealed an expansion of SEB-induced polyfunctional T cells with a low chronic activation profile in EU subjects.

Interestingly, the cohort of serodiscordant couples in this study reported an average of more than 13 years in relationships, in which the majority of HIV-infected partners were successfully treated with ART. This period is longer than the time of HIV diagnosis and treatment (10.2 years), which indicates that the uninfected partner had been highly exposed for some period while the partner had detectable viremia, before ART. During the relationship period, these serodiscordant couples have persistent despite low sexual HIV exposure.

Indeed, HIV-1 infected individuals demonstrated an increased frequency of CD4+ T cells secreting IL-17 and IL-22 upon Gag stimulation. It is possible that the increased frequency of Th22 and Th17 cells in the peripheral blood could be related to the immunological status of these individuals, who were all ART-suppressed patients, with CD4+ T cells counts of 618.2 cells/mm^3^. In addition, no correlation was verified with viral load or CD4+ T cell counts with the frequency of Th17 and Th22/Tc22 cells. Moreover, Th17 cells are a primary target during HIV infection, and their loss and dysregulation partially recovers after successful ART^22^. The depletion of Th22 cells also occurs in the intestinal mucosa of chronically infected individuals^14^. The lower counts of Th17/Th22 cells in the peripheral blood could be the result of primary CD4+ T cells depletion in the intestinal tissues. In persistently SIV-infected animals, a depletion of Th17 cells as well as Th22 cells in blood and intestine has been observed^23^. Moreover, the numbers of Th17 in blood and gut mucosal are preserved in long-term nonprogressors^24^.

Some similarities were detected between EU subjects and their HIV partners that did not distinguish between the effects of pathogen exposure and HIV infection. As we verified, EUs had higher frequencies of p15 Gag-induced CD4+ IL-22+ T cells, whereas HIV-infected subjects demonstrated a broader IL-22 response, including p15 and p24 responses. Additionally, similar IL-22 frequencies of both Gag and SEB were induced by CD8+ T cells in EU subjects and HIV partners. Notably, exposure to HIV-1 did not induce an IL-17 Gag response but was able to induce IL-22 secretion by CD4+ and CD8+ T cells. To our knowledge, there are no published data evaluating Tc22 cells in HIV-1 infection.

The role of IL-22 in EU subjects has been controversial. It has been suggested that the increased serum levels of IL-22 and acute-phase proteins in EU subjects could be associated with resistance to infection^12^. However, a lower degree of activation could be related to resistance to HIV infection in female sex workers in Kenya, who demonstrated decreased IL-22 secretion by PBMCs stimulated with SEB or left unstimulated and suppressed IL-22 expression in cervical cells^15^. In contrast, since IL-22 secretion may occur from several types of cells, we evaluated IL-22 secretion by CD4+ and CD8+ T cells, and verified an increased frequency of Th22/Tc22 induced by SEB and Gag peptides in EU subjects compared to HC. This suggest a protective role at mucosal sites. Selective loss of Th22 cells and a reduction in IL-22 cytokine secretion by CD4 T cells occurred in patients with untreated HIV-1 infection, which was restored in part by ART^25^. The loss of Th22 cells and disruption in the balance of Th22 and T regulatory cells may contribute toward systemic immune activation and mucosal immune deficiency during HIV-1 infection^25^. Considering that EU subjects are HIV-exposed but uninfected, the increment of Th22/Tc22 cells in peripheral blood may suggest a protective strategy in the HIV infection. A more detailed investigation is necessary to determine whether the presence of circulating Gag-induced IL-22-secreting T cells in EU individuals contributes to the control of HIV-1 infection.

Although we identified Gag-induced Th22 and Tc22 cells in EU subjects, polyfunctional CD4+ and CD8+ T cells were generated only upon SEB stimulation. In contrast, HIV-1 partners demonstrated expansions of both p17 and p24 Gag polyfunctional responses (4-5 combinations, including IFN-γ, IL-2, IL-17, IL-22, and MIP-1β), as expected. Polyfunctionality by CD4+ T cells in response to p17 and p24 has been associated with viremic controllers in the absence of ART^20^ and HIV nonprogressors that have demonstrated recovered polyfunctionality of CD8+ T cells for CD107a, IFN-γ, IL-2, MIP-1β and TNF-α; these observations could represent one factor that prevents disease progression^17^. Our results showed that HIV-infected individuals who are successfully ART-treated demonstrate recovered HIV Gag polyfunctional T cell function. To better verify these immunological changes, we chose to investigate couples rather than non-related HIV-infected individuals.

The robust, non-specific SEB-induced polyfunctional response of circulating CD4+ and CD8+ T cells in EU subjects, despite HIV-1 exposure, could also occur in response to exposure to other viruses, such as cytomegalovirus (CMV). In fact, previously we verified 100% frequency of CMV seropositivity in EU individuals, similarly to HIV, whereas only 65% seropositivity is found in healthy control individuals^26^. Indeed, CMV seropositive donors have higher numbers of polyfunctional CD8+ T cells associated with the expansion of polyfunctional CD8+ CD57+ T cells, indicating that CMV improves the CD8 response to SEB^27^. This increase in polyfunctionality, which can provide an immunological advantage in response to other pathogens, is attributable to CD8+ CD57+ T cell expansion in CMV-seropositive individuals^27^. We have not determined the serology for other viruses that we know are prevalent in HIV-infected individuals and can be sexually transmitted to EU partners; the question of whether SEB-induced polyfunctional T cells have a role in acute HIV infection could be interesting to explore.

Another intriguing finding in EU subjects was related to CD38 expression in polyfunctional T cells. We verified that the polyfunctionality of CD4+ and CD8+ T cells induced by Gag was observed in CD38+ cells in HIV-infected individuals, whereas in EUs, polyfunctionality was observed in CD38- T cells in response to SEB. The expression levels of CD38 in CD8+ T cells declined steadily in HIV-positive subjects after the beginning of ART but increased transiently in parallel with episodes of viral replication^28^. Usually, polyfunctionality indicates good prognosis of HIV infection, whereas we found polyfunctionality only in activated CD38+ T cells. This finding suggests that polyfunctional T cells in ART-treated HIV individuals do not reach the similar polyfunctional status as verified in the healthy control and EU groups, in which polyfunctional T cells are CD38-. Moreover, HIV controllers show CD38^−^/HLA-DR^+^ HIV-specific CD8^+^ T cell cytotoxicity^29^. Despite the lack of infection in EUs, they present low activation in CD4+ T cells^11^,^30^. Our findings indicate that this activation profile, represented by the CD38 expression marker, could be an interesting marker for monitoring HIV infection, and the absence of CD38 expression indicates low activation status in HIV-exposed individuals^11^,^30^. In this context, less activation of CD4+ T cells is associated with lower susceptibility to HIV infection. Additionally, CD38- HLA- DR+ CD8+ T cells in HIV controllers are related to a moderate state of activation that leads to a good cytotoxic response and prevents deleterious effects on immune chronic activation^29^.

Our results show that polyfunctional T cells with low activation profiles could be one of the factors associated with lack of infection in EU individuals.

## Materials and Methods

### Study subjects

HIV-1-serodiscordant couples from an outpatient clinic at the Emílio Ribas Infectious Diseases Institute in São Paulo, the Ambulatory Service of the Secondary Immunodeficiency Clinic of the Clinical Hospital, University of São Paulo Medical School (HC/FMUSP) and the Reference Center and Treatment in STD-AIDS in São Paulo, Brazil were enrolled. The EU group (n = 16) consisted of 8 males and 8 females, with a mean age of 40.6 years (range 25–57); the HIV-infected individuals (n = 15) consisted of 14 males and 1 female, with a mean age of 41.3 years (range 28–53). Healthy donors (n = 15) consisted of 8 males and 7 females, with a mean age of 37.0 years (range 27–54 years), who were seronegative for HIV-1. Homosexual (n = 7) and heterosexual couples (n = 9) reported a mean relationship duration of 13 years (range 2–26) with a single partner. Couples reported participating in vaginal, anal and oral sex, including 5 episodes of unprotected sexual intercourse at a frequency of 3–4 times per month^29^,^31^. The inclusion criteria included being over 18 years of age, reported participation in unprotected sex and having a single partner for over 1 year. Exclusion criteria included the use of immunosuppressant or immune-modifying drugs and pregnancy. The EU cohort was seronegative at the studied time point. The majority (14/15) of HIV-1-infected individuals were receiving antiretroviral therapy (ART) treatment; detectable viral loads (VLs) were verified in 5/15 individuals (51, 181, 16,561, 17,511 and 50,930 copies of RNA/mL). The average absolute CD4 count for the EU group was 1,234 ± 85.7 cells/mm^3^ (mean ± SEM), whereas that of the HIV-infected group was 618.2 ± 94.1 cells/mm^3^ and that of the healthy control group was 969.4 ± 68.9 cells/mm^3^. This study was approved by the São Paulo University Institutional Use Committee (10459312.8.3001.0061) and informed consent was obtained from all subjects. All experimental protocols within this study were performed in accordance with the Ethics Committee of this institution.

### Cell culture and flow cytometry

Peripheral blood mononuclear cells (PBMCs) were isolated from heparinized venous blood via Ficoll-Hypaque gradient centrifugation (GE Healthcare Bio-Sciences AB, Uppsala, Sweden) and were diluted in RPMI medium supplemented with 10% AB human serum (Sigma, St. Louis, MO, USA). Cultures consisting of 1.5 × 10^6^ cells/mL were incubated in 48-well plates (Costar, Cambridge, MA, USA) in the absence of a stimulus or with 1 μg/mL SEB (Sigma), 5 μg/mL of 3 pools of HIV-Gag peptides (15-mers of the HIV-1 HXB2 Gag peptide, with overlaps of 11 residues; NIH AIDS Research and Reference Reagent Program) or CD107a PE-Cy5 Ab (5 μL, BD Biosciences, San Jose, CA, USA) for 6 h at 37 °C in 5% CO_2_. The Gag peptides (a total of 123 peptides) were divided into 3 groups according to the amino acid sequence alignments for the p17 Gag region (Gag1, 33 peptides, 7872–7904), the p24 Gag region (Gag2, 59 peptides, 7904-7962) and the p15 Gag region (Gag 3, 33 peptides, 7962-7994). After 2 h of incubation, Brefeldin A (10 μg/mL, Sigma) was added to the cultures, and the cultures were incubated for another 4 h.

After incubation, the cells were washed and incubated with LIVE/DEAD Fixable Red Dead Cell Stain Kit (Invitrogen, Carlsbad, CA, USA) for 30 min at room temperature, followed by fixation with Cytofix/Cytoperm solution (BD Bioscience) for 20 min and permeabilization with Perm/Wash solution for 20 min at 4 °C. The cells were then stained with CD3 BV605 (SK7), CD4 V500 (RPA-T4), CD8 PerCP-cy5.5 (RPA-T8), CD38 Alexa Fluor 700 (HIT2), IFN-γ V450 (B27), IL-2 Pe-Cy7 (MQ1-17H12), MIP-1β APC-H7 (D21-1351), IL-17a Alexa Fluor 488 (eBiosciences, San Diego, CA, USA), IL-22 PE, (eBiosciences) and Granzyme B V450 (GB11); all antibodies were purchased from BD Biosciences (San José, CA, USA). Next, the samples were washed with Perm/Wash buffer (BD Biosciences) and diluted in isotonic solution. A total of 500,000 events were collected and analyzed by flow cytometry (LSR Fortessa, BD Biosciences, USA) using FACS-Diva (BD Bioscience) and FlowJo 10.0.6 (Tree Star, Ashland, OR, USA) software programs. Fluorescence Minus One (FMO) controls were performed for all antibody panels to confirm proper compensation and define positive signals. Boolean gate arrays were created using FlowJo software. These analyses determined the expression frequency of each cytokine based on all possible combinations of the 5 different cytokines. Analysis of polychromatic flow cytometry data was performed with the SPICE Program (version 2.9, Vaccine Research Center, NIAID, USA).

### Statistical analysis

All cytokine measurements were background subtracted, taking into account the frequency of cells producing cytokines in the absence of antigenic stimulation. Kruskal–Wallis tests with Dunn’s post-tests were used to evaluate differences among HIV-infected individuals, EU subjects and healthy controls. p ≤ 0.05 was considered statistically significant.

## Additional Information

**How to cite this article**: Oliveira, L. M. S. *et al.* Increased frequency of circulating Tc22/Th22 cells and polyfunctional CD38^–^ T cells in HIV-exposed uninfected subjects. *Sci. Rep.* **5**, 13883; doi: 10.1038/srep13883 (2015).

## Supplementary Material

Supplementary Information
