# Supplementary material for: Increased frequency of circulating Tc22/Th22 cells and polyfunctional CD38− T cells in HIV-exposed uninfected subjects
Source: Sci Rep. 2015 Sep 8;5:13883. doi: 10.1038/srep13883 (PMC4561954; doi:10.1038/srep13883)
Supplement: Supplementary Information [file srep13883-s1.pdf]

Title: Increased frequency of circulating Tc22/Th22 cells and polyfunctional CD38$^-$ T cells in HIV-exposed uninfected subjects

Authors: Luanda M. S. Oliveira[1], Josenilson F. Lima[1], Cesar A.C. Cervantes[1], Jorge Simão Casseb[1,2], Marcelo Mendonça[3], Alberto J.S. Duarte[1], Maria N. Sato[1].

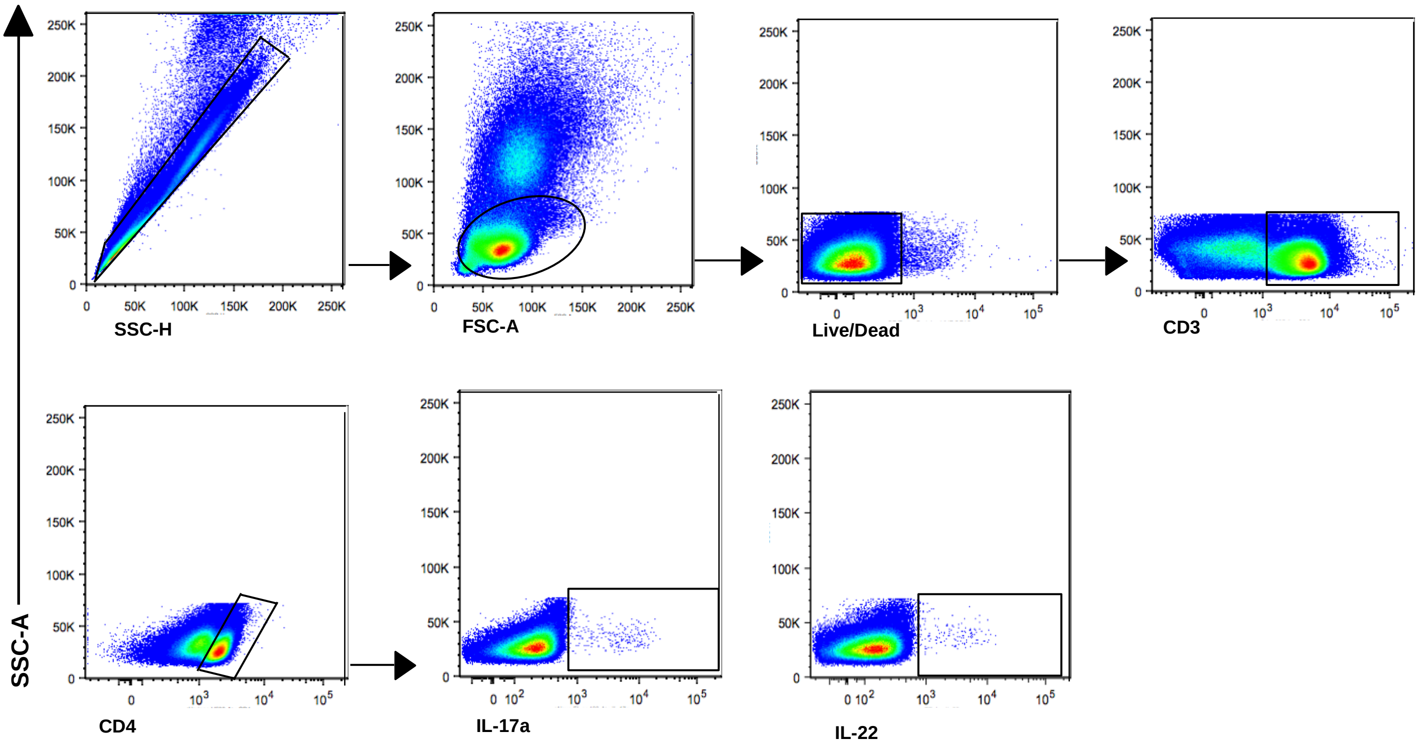

Supplementary Figure 1. Gating strategy to evaluate CD4+ T cells secreting cytokines**.** The initial gate utilized was side scatter area (SSC-A) versus side scatter height (SSC-H) to exclude doublets. Next, lymphocytes were selected in the SSC-A versus forward scatter area (FSC-A), and dead cells were excluded with LIVE/DEAD staining. CD3+ T cells were selected, and CD4+ T cells secreting IFN-γ, IL-17a and IL-22 were individually assessed.

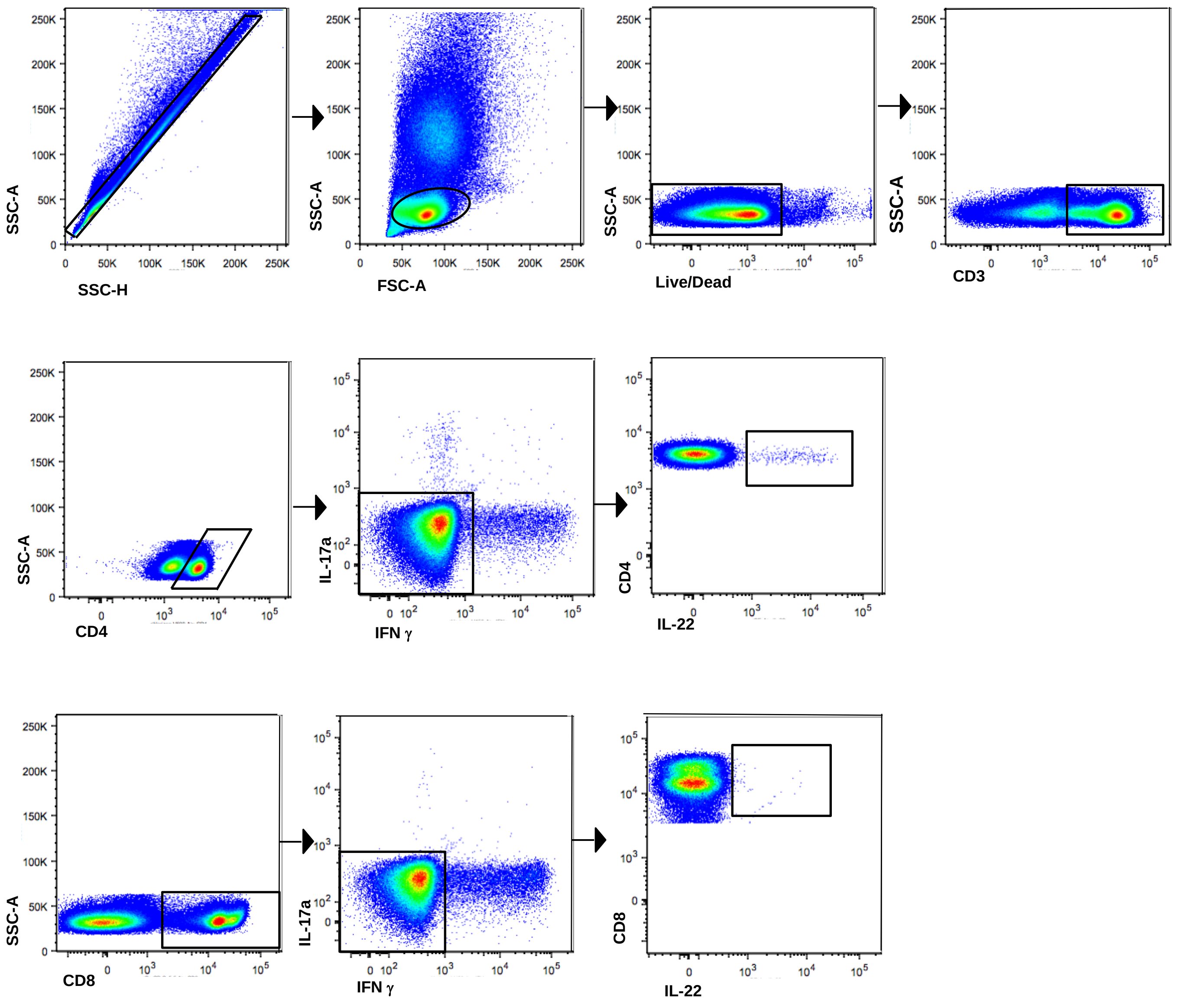

Supplementary Figure 2. Gating strategy to evaluate Th22 and Tc22 cells**.** The initial gate utilized was side scatter area (SSC-A) versus side scatter height (SSC-H) to exclude doublets. Next, lymphocytes were selected in the SSC-A versus forward scatter area (FSC-A), and dead cells were excluded with LIVE/DEAD staining. CD3+ T cells were selected, and CD4+ or CD8+ T cells were also selected, excluding IFN-γ and IL-17a, and were evaluated for IL-22 production.

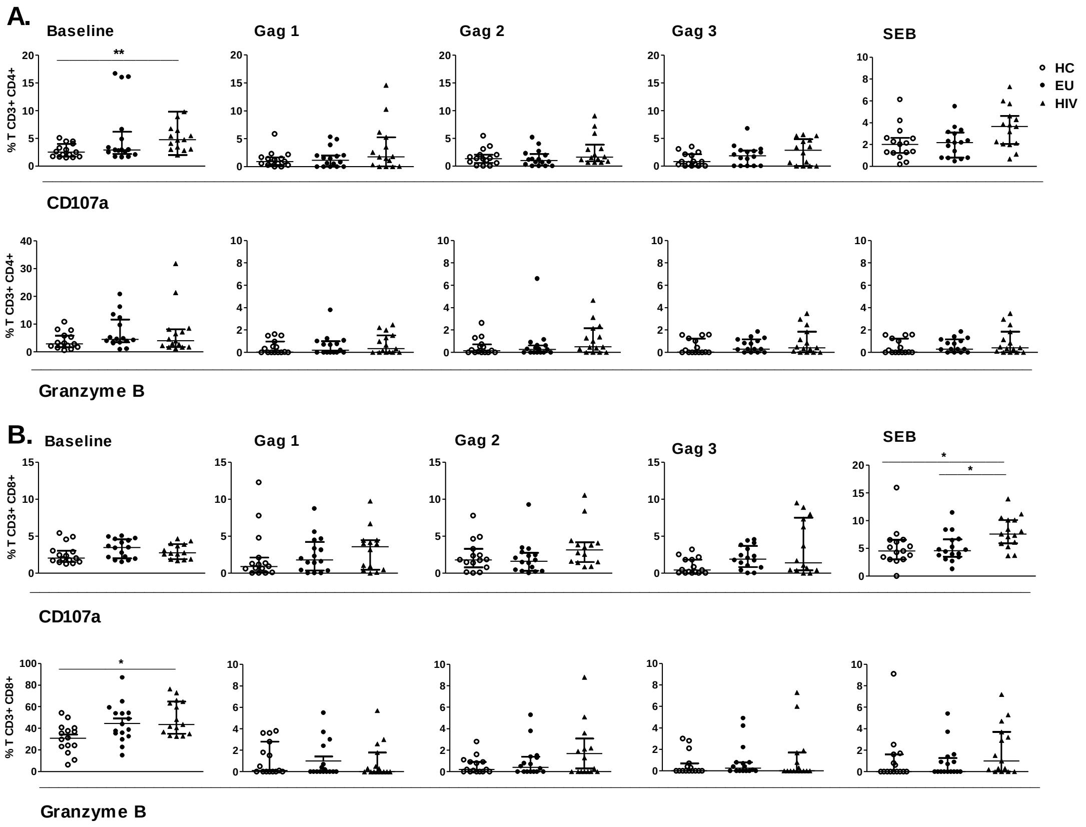

Supplementary Figure 3. Cytotoxic CD4+ and CD8+ T cells in EUs and HIV-infected subjects**.** PBMCs from HCs (n=15), EUs (n=16) and HIV-infected individuals (n=15) were cultivated with medium (baseline), HIV Gag peptide pools[Gag1 (p17), Gag2 (p24), and Gag3 (p15)], or SEB for 6 h and Brefeldin A for 4 h. TCD4+ (A) and TCD8+ (B) cells expressing CD107a and granzyme B were assessed by flow cytometry. Frequency of CD4+ or CD8+ T cells was subtracted from baseline. The results are expressed **as medians and IQRs**. *p≤0.05and **p≤0.01.

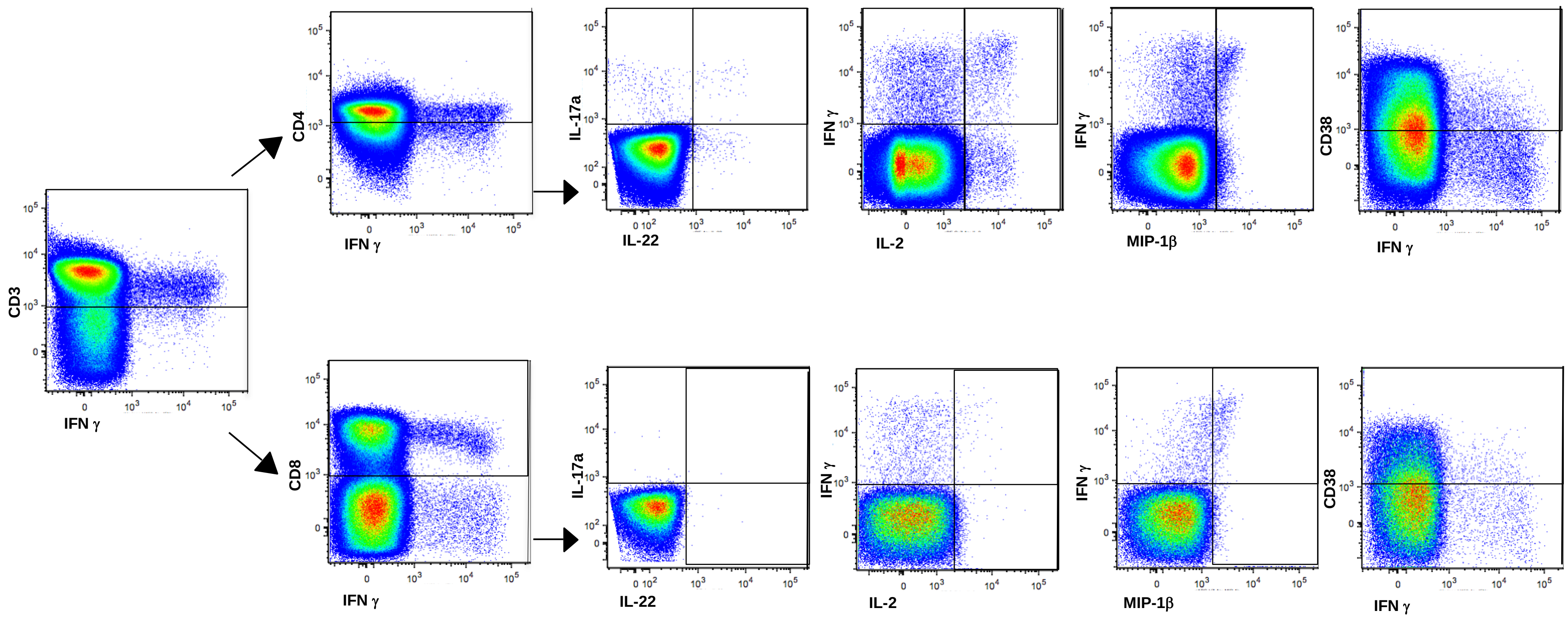

Supplementary Figure 4. Gate strategy for polyfunctional CD4+ and CD8+ T cells. The expression of each cytokine was selected for in CD3+ T cells, followed by CD4+ T or CD8+ cells in the same combination. Next, Boolean evaluation of several combinations of secreted cytokines was performed, and CD38 expression was assessed.
